# Laboratory Evaluation of Five Chitin Synthesis Inhibitors Against the Colorado Potato Beetle, *Leptinotarsa decemlineata*

**DOI:** 10.1673/031.007.5001

**Published:** 2007-09-27

**Authors:** R. Karimzadeh, M. J. Hejazi, F. Rahimzadeh Khoei, M. Moghaddam

**Affiliations:** ^1^ Department of Plant Protection, College of Agriculture, University of Tabriz, Tabriz, 51666–14888, Iran; ^2^ Department of Agronomy and Plant Breeding, College of Agriculture, University of Tabriz, Tabriz, 51666–14776, Iran

**Keywords:** cyromazine, benzoylphenyl ureas, diflubenzuron, hexaflumuron, lufenuron, triflumuron

## Abstract

Results of laboratory experiments are reported that tested the effects of five chitin synthesis inhibitors, diflubenzuron, cyromazine, lufenuron, hexaflumuron and triflumuron. on second instars of the Colorado potato beetle, *Leptinotarsa decemlineata* (Say) (Coleoptera: Crysomelidae), originally collected from potato fields of Bostanabaad, a town 66 km southeast of Tabriz, Iran. In bioassays, the larvae were fed potato leaves dipped in aqueous solutions containing chitin synthesis inhibitors. The mortalities and abnormalities of the treated larvae were recorded 72 hours after treatments. LC_50_ values were 58.6, 69.6, 27.3, 0.79 and 81.4 mg ai/ L for diflubenzuron, cyromazine, lufenuron, hexaflumuron and triflumuron, respectively. Compared with phosalone, which is one of the common insecticides used for controlling this pest in Iran, lufenuron and hexaflumuron seem to be much more potent, and if they perform equally well in the field, they would be suitable candidates to be considered as reduced risk insecticides in management programs for *L. decemlineata* due to much wider margin of safety for mammals and considerably fewer undesirable environmental side effects.

## Introduction

*Leptinotarsa decemlineata* (Say) (Coleoptera: Crysomelidae), the Colorado potato beetle is the most devastating defoliator of potato plants worldwide. If populations of this pest are not controlled, they can cause a total loss of yield by defoliating potato plants prior to tuber formation. Tuber formation and filling are the most susceptible stages of potato plants with regard to damage by *L. decemlineata*. Therefore, pest management guidelines are designed to limit total defoliation to 10–25% at these stages (Hare 1990).

Widespread resistance of *L. decemlineata* to most insecticide chemical groups has been reported in many parts of the world (Harris and Svec 1981; French *et al*. 1992). In some parts of the United States of America this insect has even developed resistance to the newly commercialized neonicotinoids (Mota-San chez *et al*. 2006). Effective chemical control of this pest requires new insecticides with novel mechanisms of action. The effects of chitin synthesis inhibitors on *L. decemlineata* have been studied by several researchers (Cutler *et al*. 2005a; Furlong and Groden 2001; Sirota and Grafius 1994). Cutler *et al.* 2007 reported that novaluron at 50 g ai/ha provided excellent and prolonged effect against *L. decemlineata* and could be a valuable tool in future *L. decemlineata* management programs. These insecticides with specific mode of action are relatively new to Iran. These compounds interfere with formation of chitin and control immature stages of many pests with relatively low harm to beneficial arthropods (Consoli *et al*. 2001; Wakgari and Giliomee 2003; Catangui *et al*. 1996). Based on the study conducted by Lucas *et al*. 2004 cyromazine was not lethal to the first and third instars of the lady beetle, *Coleomegilla maculata* (a predator of *L. decemlineata*) and seems to be a suitable compound for protection of *C. maculata* populations.

Consoli *et al*. 2001 stated that lufenuron and triflumuron did not affect the parasitization capacity of the parasitoid, *Trichogramma galloi*, and caused almost 100% mortality of larvae of the sugar cane borer, *Diatraea saccharalis*, when used to treat eggs prior to parasitization. Laboratory bioassays indicated that triflumuron did not cause significant mortality in *Coccidoxenoides peregrinus*, a parasitoid of the mealybug, *Pseudococcus longispinus* (Wakgari and Giliomee 2003). Most of these compounds have low soil persistence and high residual activity on foliage (Cutler *et al*. 2005b; Malinowski and Pawinska 1992; Tuttle and Ferro 1988). Thus, the use of these insecticides may allow build up of the population of at least some natural enemies compared with chemical insecticides of broader spectrum action.

The aim of this study was to assess the effects of some chitin synthesis inhibitors against *L. decemlineata* population from an important potato-growing region of Iran by estimating their LC_50_ values.

## Materials and Methods

### Insecticides

The chitin synthesis inhibiting insecticides used were: diflubenzuron (25 WP, Hebei vian biochemical, www.chinanusa.net), hexaflumuron (Consult 10 EC, Dow AgroSciences, www.dowagro.com); triflumuron (Starycide 48 SC, www.bayer.com), lufenuron (Match 50 EC, www.syngenta.com). These compounds belong to benzoylphenyl ureas structurally. The other insecticide tested was cyromazine (Trigard 75 WP, Novartis, www.novartis.com) which belongs to a different chemical group, thetriazines.

### Insects

The *L. decemlineata* colony was established using adults and eggs collected from potato fields in Bostanabaad and maintained in greenhouse conditions (26 ± 3° C; RH = 50 ± 15%; photoperiod of 16:8 L:D). The 2^nd^ instars of the colony reared over three generations were used to test the insecticides. To have uniformly aged larvae in the experiments, the 1^st^ instars that were ready to molt were separated 24 hours prior to the bioassays and newly (up to 24 hours) molted 2^nd^ instars were used.

### Bioassays

Based on preliminary experiments, the ranges of concentrations tested were 62.5–168, 22.5–450, 25–50, 0.5–1.6 and 50–75 mg ai/L for triflumuron, cyromazine, lufenuron, hexaflumuron and diflubenzuron, respectively. Each treatment consisted of five concentrations and a control. Potato leaves were dipped in aqueous solutions with different concentrations of the chitin synthesis inhibitors. Tween 80 was used at a concentration of 500 ppm as a surfactant to ensure complete wetting of the leaves. After drying, the petiole of each leaf was inserted in a 1.5 ml micro tube filled with tap water through a hole made in the micro tube cap. Each micro tube was then put in a 14 × 7 × 4 cm transparent plastic box. Fifteen 2^nd^ instars (up to 24 hours old) of *L. decemlineata* were put on each treated leaf. One screened hole (4 cm in diameter), on the removable lid of each plastic box provided ventilation. The boxes were kept in insectarium at 25 ± 1° C, RH = 50 ± 10% and a photoperiod of 16:8 L:D. The mortalities and abnormalities of the treated larvae were recorded 72 hours after treatment. This was done because the larvae in the controls molted to 3^rd^ instar after this period of time. The treatments were replicated four times. Each replication was done at a different day and the solutions used for each treatment were freshly prepared each time.

**Figure 1.**
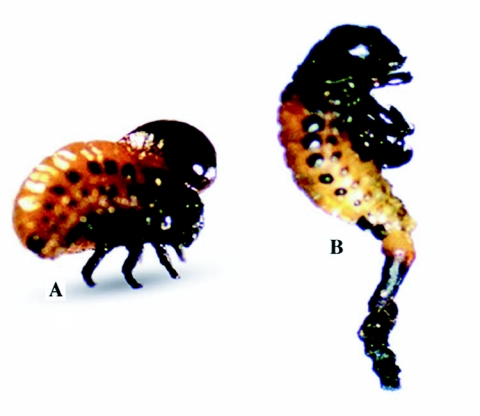
Two 2^nd^ instars of *Leptinotarsa decemlineata* 72 hours after treatment with Cyromazine. A blister filled with hemolymph protruding from prothorax (A) and the hindgut everted from the anus (B).

The probit option of the Statistical Package for Social Sciences was used for data analysis (SPSS 1999). The toxicities of the insecticides tested were evaluated based on 95% confidence limits of LD_50_ ratios. If the 95% confidence interval included 1, then the difference between LC_50_s were considered insignificant (Robertson and Preisler 1992).

## Results and Discussion

### Symptoms

Chitin synthesis inhibitors act by interfering with chitin synthesis. Hence, most of the larvae treated with these compounds showed symptoms at molting. Partial molting was seen in some larvae treated with benzoylphenyl ureas. Inability in casting the old head capsule occurred in others. This was followed by death of the larvae.

The toxicity symptoms in larvae treated with cyromazine were most dramatic and seen as elongation and increase in turgor. The intersegmental membranes were stretched. In some treated larvae hindgut protruded from the anus or a blister filled with brown fluid (probably hemolymph) was seen on the first tergum (Figure 1). Hughes *et al*. 1989 also reported these symptoms. These researchers suggest that cyromazine probably decreases chitin synthesis in endocuticle of the hindgut and weakens the junction of the midgut and hindgut. The poisoned larvae became dark red in color and were lethargic, while untreated larvae had a shiny bright red appearance. The blister-like lesions and lethargy shown by the 2nd instars of *L. decemlineata* in this study were similar to those reported by Sirota and Grafius (1994). The symptoms exhibited by the treated *L. decemlineata* larvae were consistent with symptoms reported for some other species of insects such as *Lucilia cuprina, Manduca sexta* and *Lymantria dispar* treated with chitin synthesis inhibitors (Abdel-Monem *et al*. 1980; Kotze *et al*. 1992; Root and Dauterman 1996). The similarities in symptoms seen in different orders of insects probably indicate a common mode of action (Sirota and Grafius 1994).

**Figure 2.**
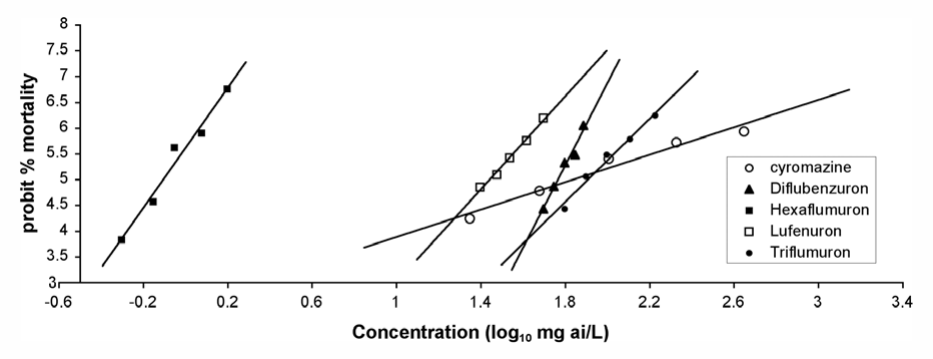
Concentration- response relationship between chitin synthesis inhibitors and the 2^nd^ instars of *Leptinotarsa decemlineata*, 72 hours after treatment.

### LC_50_ values

LC_50_ values for the 2^nd^ instars of the *L. decemlineata* are shown in Table 1. Figure 2 presents the relationship between the probit of percentage mortalities and the logarithm of the concentrations of the chitin synthesis inhibitors tested. Hexaflumuron and lufenuron were more effective than the other insecticides. Based on the estimated LC_90_, the toxicities of the chitin synthesis inhibitors tested can be rated in the following order: hexaflumuron> lufenuron> diflubenzuron> triflumuron> cyromazine.

**Table 1.**
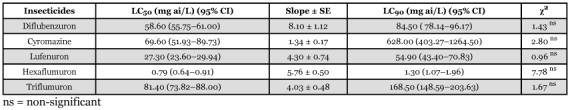
LC_50_ values of CSI s on 2nd instars of *Leptinotarsa decemlineata* 72 hours after using the leaf dip method.

Triflumuron was the least toxic to the 2nd instars compared with the other benzoylphenyl ureas tested. Malinowski and Pawinska (1992) studied the effect of chlorfluazuron, teflubenzuron, hexaflumuron, triflumuron and GR 572 on *L. decemlineata*. They also reported that triflumuron was less effective than other benzoylphenyl ureas. Based on the 95% confidence intervals of the LC_50_ ratios, the different insecticides were compared with each other. Toxicities of the CSIs tested were significantly different except that the difference between diflubenzuron and triflumuron was not significant. The LC_50_ value for triflumuron reported by Cooper *et al*. (1983) was 75 mg ai/L for 2nd instars of *L. decemlineata*, which compares favorably with our estimate of 81.4 mg ai/L. This may be due to the relative similarities in sensitivities of the populations tested and the experimental procedures used. Furlong and Groden (2001) reported 123 mg ai/L as the LC_50_ of cyromazine for 2nd instars of *L. decemlineata*, which was larger than 69.6 mg ai/L in our study. In their study the larvae fed on treated leaf disks for 24 hours, while in ours the feeding period on treated leaves was 72 hours. The LC_50_ of diflubenzuron in our study (58.6 mg ai/L) is comparable to 50 mg ai/L reported by Hegazy *et al*. (1989).

The slopes of the dose-response lines of the chitin synthesis inhibitors tested (except for cyromazine) were quite steep and the differences between the highest and lowest concentrations were low. That is, the population is phenotypically homogeneous, and with a fairly small increase in insecticide concentration, the mortality would increase considerably. This necessitates more careful use of these chitin synthesis inhibitors in the field to prevent exerting a high selection pressure that could eliminate the susceptible individuals and lead to selection of resistant individuals (Robertson and Preisler 1992). Cyromazine is currently proposed for control of Colorado potato beetle and leafminers on potato (Anonymous 1999). This insecticide provided good control of early instars of *L. decemlineata* in field trials as reported by Bishop *et al*. (1990) and Sirota and Grafius (1994).

Our results indicated that among the chitin synthesis inhibitors tested, hexaflumuron and lufenuron (with LC_50_ values of 0.79 and 27.3 mg ai/L respectively) were the most effective at low concentrations against the *L. decemlineata*. In comparison, phosalone, which is one of the most commonly used insecticides for controlling this pest in Iran, had an LC_50_ range of 48.72 – 64.12 mg ai/L for five Iranian populations of *L. decemlineata* tested (Pourmirza 2005). Hence, these chitin synthesis inhibitors seem to be more potent and, if they perform equally well in the field, they would be suitable candidates to be considered as reduced risk insecticides for controlling *L. decemlineata* in Iran due to their relatively much wider margin of safety (Catangui *et al*. 1996; Griffith *et al*. 2000; Tunazand Uygun 2004). Since these compounds do not belong to the groups of insecticides conventionally used for *L. decemlineata* control in Iran, they can be used in rotation with other insecticides. This would conform to the effective *L. decemlineata* management strategy of not applying the same class of compound more than once within a growing season.
